# A Longitudinal Study of Stress During Pregnancy, Children’s Sleep and Polygenic Risk for Poor Sleep in the General Pediatric Population

**DOI:** 10.1007/s10802-023-01097-2

**Published:** 2023-07-13

**Authors:** Desana Kocevska, Isabel K. Schuurmans, Charlotte A. M. Cecil, Pauline W. Jansen, Eus J. W. van Someren, Annemarie I. Luik

**Affiliations:** 1 Department of Sleep and Cognition, Netherlands Institute for Neuroscience, Amsterdam, Netherlands; 2 Department of Child and Adolescent Psychiatry/Psychology, Erasmus MC University Medical Center, Rotterdam, Netherlands; 3 Generation R Study, Erasmus MC University Medical Center Rotterdam, Rotterdam, Netherlands; 4 Department of Epidemiology, Erasmus MC University Medical Center Rotterdam, Rotterdam, Netherlands; 5 Department of Psychology, Erasmus University Rotterdam, Education, and Child Studies, Rotterdam, The Netherlands; 6 Department of Psychiatry, UMC, Amsterdam Public Health Research Institute and Amsterdam Neuroscience Research Institute, Vrije Universiteit, Amsterdam, Netherlands; 7 Department of Integrative Neurophysiology, Center for Neurogenomics and Cognitive Research, Vrije Universiteit Amsterdam, Amsterdam Neuroscience, Netherlands

**Keywords:** Prenatal Stress, Sleep Problems, Sleep Duration, Polygenic Risk Score

## Abstract

Early life stress is robustly associated with poor sleep across life. Preliminary studies suggest that these associations may begin already in utero. Here, we study the longitudinal associations of prenatal psychosocial stress with sleep across childhood, and assess whether prenatal stress interacts with genetic liability for poor sleep.

The study is embedded in the Generation R population-based birth cohort. Caregivers reported on prenatal psychosocial stress (life events, contextual, parental or interpersonal stressors) and on children’s sleep at ages 2 months, 1.5, 2, 3 and 6 years. The study sample consisted of 4,930 children; polygenic risk scores for sleep traits were available in 2,063.

Prenatal stress was consistently associated with more sleep problems across assessments. Effect sizes ranged from small (B = 0.21, 95%CI: 0.14;0.27) at 2 months to medium (B = 0.45, 95%CI: 0.38;0.53) at 2 years. Prenatal stress was moreover associated with shorter sleep duration at 2 months (B_hrs_ = -0.22, 95%CI: -0.32;-0.12) and at 2 years (B_hrs_ = -0.04, 95%CI -0.07; -0.001), but not at 3 years (B_hrs_ = 0.02, 95%CI: -0.02;0.06). Prenatal negative life events interacted with polygenic risk for insomnia to exacerbate sleep problems at 6 years (B_interaction_ = 0.07, 95%CI: 0.02;0.13).

Psychosocial stress during pregnancy has negative associations with children’s sleep that persist across childhood, and are exacerbated by genetic liability for insomnia. Associations with sleep duration were more pronounced in infancy and seem to attenuate with age. These findings highlight the role of the prenatal environment for developing sleep regulation, and could inform early intervention programs targeting sleep in children from high-risk pregnancies.

**Supplementary Information:**

The online version contains supplementary material available at 10.1007/s10802-023-01097-2.

## Introduction

Sleep problems in early childhood are common (~ 30% up to age of 5 years Byars et al., 2012; Owens et al., 2000), and are associated with a myriad of adverse mental health and developmental outcomes (from externalizing and externalizing problems to autism and ADHD). Therefore, it is becoming increasingly important to identify early determinants of sleep problems (Chatterjee et al., 2018; Morales-Muñoz et al., 2018; Palagini et al., 2015). Childhood sleep is shaped by a number of biopsychosocial factors, including: a) intrinsic, biological characteristics such as sex and genetic predisposition (Kocevska et al., 2021; Mallampalli & Carter, 2014) extrinsic, environmental factors such as sociodemographic characteristics and parental stress (Sorondo & Reeb-Sutherland, 2015); and c) their interplay, through gene-by-environment interactions and underlying molecular mechanisms such as epigenetics (Koopman-Verhoeff et al., 2020). Identifying environmental factors that predispose children to poor sleep is of particular interest, as changing environmental characteristics is most often more tangible than targeting the underlying biology. Importantly, sleep can serve as a potentially modifiable factor to improve mental health across childhood and beyond.

Early life stress, particularly during sensitive developmental periods, may alter sleep regulation, resulting in poor sleep from childhood into adulthood (Lo Martire et al., 2020). The earliest determinants of child sleep disturbance can likely be found already in utero (Pennestri et al., 2021). Most consistently, maternal symptoms of mental disorders during pregnancy, and in particular depressive and anxiety symptoms, have been associated with poorer sleep in infancy (3 months) (Morales-Muñoz et al., 2018) and early childhood (up to 3 years) (O'Connor et al., 2007; Toffol et al., 2019). In addition, multiple studies have assessed broader measures of psychosocial stress during pregnancy showing negative associations with children’s sleep across childhood (Ksinan Jiskrova et al., 2021; Simcock et al., 2019; van den Heuvel et al., 2021). Ksinan Jisrkrova et al. (2021) found that the occurance of stressful life events in 3,272 pregnant mothers are associated with more sleep problems from 1.5 to 11 years, though effect estimates were small (estimate = -0.1). In addition, Simock et al. (2019) reported that maternal negative affect is associated with more sleep problems at 2.5 and 4 years of age (n = 134 and 118, respectively). One study of 594 mother–child dyads, however, showed no associations of self-perceived stress, family crises, negative life events or cortisol secretion patterns during pregnancy with objectively assessed sleep at 4–6 years of age (Chatterjee et al., 2018). Though the studies conducted thus far indicate that childhood sleep is affected by a stressful prenatal environment, some important gaps remain. First, studies have mainly focused on maternal mental disorders or self-perceived stress scores assessed with a single questionnaire, not taking into account different domains of stress. Second, associations of prenatal stress with different aspects of children’s sleep (e.g., duration and quality) and across different developmental periods (e.g. infancy and childhood) have not been assessed simultaneously. Finally, studies thus far have not addressed any potential interaction between genetic predisposition and environmental effects in the association between prenatal stress and childhood sleep.

Within the current study we investigated the prenatal environment by quantifying stressors during pregnancy across multiple domains, including negative life events (e.g., death in family), contextual stressors (e.g., poor housing conditions, financial difficulties), parental stressors (e.g., parental psychopathology, substance abuse), and interpersonal stressors (e.g., family relationship difficulties). Moreover, we studied both qualitative aspects of sleep (insomnia-like problems) as well as sleep duration, which were both reported by the caregiver between 2 months and 6 years of age. Importantly, we corrected all analyses for important confounders, including perinatal factors, socio-demographic characteristics, but also prenatal tobacco exposure and postnatal depressive symptoms of the mother. Finally, we ascertained if genetic predisposition for poor sleep modifies the effect of prenatal stress on sleep across childhood. We hypothesized that early life stressors will be associated with poor sleep across childhood and these effects will be cumulative, not domain specific. In addition, we hypothesized that associations will be strengthened in the presence of high polygenic risc for insomnia.

## Methods

### Participants

Information from children and their caregivers was obtained from The generation R Study, a population-based prospective cohort from fetal life onwards (Kooijman et al., 2016). In short, pregnant mothers from Rotterdam, The Netherlands, were eligible to enroll if they had a delivery date between 2003-2006. In total, 9,778 mothers were enrolled. The study sample is largely representative of the underlying population of the Netherlands, although included participants were more likely to have a higher educational level and income. Mothers, their partners, and their children took part in a diverse array of assessments, including behavioral, cognitive, and socio-demographic measures.

We excluded children if prenatal stress scores could not be computed due to > 50% frequencies of missing items (n = 2,590, 26%), or when sleep assessments were missing at 2 or more time points (n = 2,381, 32.5%). The final sample included 4,930 children. In a subsample of genotyped children of European Ancestry (n = 2,063) effect modification of prenatal stress with genetic risk were assessed.

The general design, research aims, and specific measurements of The Generation R Study have been approved by the Medical Ethical Committee of Erasmus MC University Medical Center, in accordance with the Declaration of Helsinki of the World Medical Association. Written informed consent was obtained from parents on behalf of the child.

### Prenatal Stress

The cumulative prenatal stressors score (hereafter “prenatal stress”) included 45 items measured during pregnancy. In line with previous work (Cecil et al., 2014), we derived single prenatal stress items (coded as yes or no) from a broad range of self-report questionnaires completed by primary caregivers and partners (Online Resource 1: Supplemental Methods). These items were summed into four individual prenatal stress domains: i) life events (e.g. death in the family), ii) contextual stressors (e.g. poor housing conditions, financial difficulties), iii) parental stressors (e.g. parental psychopathology, teenage parenthood), iv) interpersonal stressors (e.g. family relationship difficulties). We computed a total prenatal stress score by summing all individual items. This method has been initially developed in a birth cohort from the UK (Cecil et al., 2014), and was replicated in the Generation R Cohort Study, showing its validity. Model fit indices showed a good model fit (Schuurmans et al., 2022).

### Sleep

We repeatedly assessed sleep problems at children’s age 2 months, 1.5, 2, 3 and 6 years. For the ages 1.5, 3 and 6 years mothers answered seven questions about sleep problems from the Child Behavior Checklist (CBCL 1.5–5) on a three-point Likert scale (0-not true, 1-somewhat true, and 2-very true (Achenbach TM, 2000). At age 2 months and 2 years, age-appropriate items on sleep problems that closely resemble the CBCL sleep items were assessed. A sleep problems total score was calculated at each time point by summing five items (Kocevska et al., 2017). The items include: ‘Doesn’t want to sleep alone’; ‘Has trouble getting to sleep’; ‘Resists going to bed at night’; ‘Sleeps less than most kids during day and/or night’; and ‘Wakes up often at night’. The sum score at 2 months did not include the ‘Resists going to bed at night’ item, and at 2 years the score additionally included the ‘Where does your child generally fall asleep?’ item. *Moreover, we studied both qualitative aspects* ranged between 0.61 and 0.74, and confirmatory factor analyses indicated that the same construct was measured over time (Kocevska et al., 2017). 

Nighttime sleep duration was calculated as the difference between caregiver-reported usual bedtimes and wake times of the child at 2 months, 2, and 3 years of age were reported. Daytime sleep duration was assessed categorically with answering options ranging from < 30 min to > 2.5 h. For each time point, sleep duration was calculated as hours of sleep per 24 h by adding nighttime and daytime sleep.

### Polygenic Risk Scores

In the generation R, DNA samples were collected from cord blood or by venipuncture at 6 years of age. Individuals were genotyped using Illumina HumanHap 610 or 660 – single nucleotide polymorphism (SNP) arrays depending on collection time (Illumina, San Diego, CA). Others have previously described genotyping and quality control steps in (Medina-Gomez et al., 2015). To avoid population stratification, for genetic analyses we included only participants of European ancestry based upon the first 4 principal components inside the range of the HapMap Phase II Northwestern European founder population. To correct for further population stratification, we calculated genetic principal components using principal component analysis (EIGENSOFT) and included them in regression models. We used imputed genotype data that passed quality control to compute PRSs based on the largest GWAS of insomnia (n = 1,331,010 participants, (Jansen et al., 2019) and sleep duration (n = 446,118,(Dashti et al., 2019) to date. Polygenic Risk Score for Insomnia (PRS_insomnia_) and Polygenic Risk Score for Sleep Duration (PRS_Sleep Duration_) were created using PRSice-2 (Choi et al., 2020). This software calculates individual PRSs by summing up all the SNP alleles associated with a trait carried by the participants weighted by the SNP allele effect size estimated in a previous GWAS. Polygenic scoring was performed in clumped variants according to linkage disequilibrium using an r^2^ < 0.10 cutoff within a 300-kb window. We calculated PRS_insomnia_ and PRS_Sleep Duration_ based on a genome-wise significant p-value threshold pT < 5e.08. Higher scores of PRS_insomnia_ indicate higher risk for insomnia, whereas higher PRS_Sleep Duration_ indicate higher genetic predisposition for longer sleep duration. PRS were standardized to a mean of 0 and a standard deviation of 1.

### Covariates

We took multiple covariates into account (Pennestri et al., 2021). Gestational age, and sex of the infant were obtained from community midwife and hospital registries at birth. Age of the child was obtained in the respective questionnaires at each time point. Ethnic origin of the child was obtained by self-report during pregnancy, being categorized according to the classification of Statistics Netherlands (2004), into ‘Western’ (European, North-American, and Oceanian) and ‘non-Western’ (Turkish, Moroccan, Indonesian, Cape Verdean, Surinamese, and Antillean). History of tobacco smoking was obtained by postal questionnaire in early, mid- and late pregnancy and categorized into: “never smoked”, “stopped smoking when pregnancy was known” and “continued smoking during pregnancy.” Postnatal depressive symptoms of the mother were assessed with the Dutch version of the Brief Symptom Inventory completed by the mother at child’s age 3 years, with internal consistency of the depression scale was α = 0.80 (Derogatis & Melisaratos, 1983; El Marroun et al., 2016).

### Multiple Imputation Technique

Frequency of missing values within the individual prenatal stress items ranged between 0.2% and 32.2% (M = 7.3%), with 54.1% having complete prenatal information. Missing values in sleep items ranged between 0 and 82% (M = 14.50%), with 41% having complete information. Therefore, after excluding participants with too much missing information (> 50% for prenatal stress and > 25% for sleep), we imputed missing items with Multivariate Imputation by Chained Equations (mice (van Buuren & Groothuis-Oudshoorn, 2011) using 30 imputed datasets and 60 iterations. Our imputation strategy was based on Van Buuren (2018). First, domain sum scores (i.e. life events, contextual stressors, parental stressors and interpersonal stressors) and sleep problems scores were passively imputed, meaning that for each completed imputed dataset sum scores were computed using complete data on individual items. Subsequently, missing items were imputed using the following information: (i) items specific to the sum score (i.e. items within the prenatal stress domain or items within the sleep score at respective time point), (ii) prenatal stress domain sum scores, (iii) the sleep scores at each time point, and (iv) auxiliary variables (Van Buuren, 2018), including variables related to the mother (age, BMI, marital status, low education, smoking, parity), as well as both mother and partner (depressive symptoms at child’s age 3 years), and child (gestational age, birth weight, ethnicity, sex).

### Statistical Analyses

Statistical analyses were performed using R version 3.6.3. First, we performed a nonresponse analysis, comparing children included in our study with those excluded due to lack of sufficient outcome data on several variables including sex, ethnicity, educational level of the mother, as well as prenatal stress scores. For our main analyses, we examined associations of the cumulative prenatal stress scores (total score and individual domain scores in separate models) with the sleep problems scores and sleep duration at each time point separately. In addition, we used a Linear Mixed Model with a random intercept and a random effect for age, to examine associations between prenatal stress and trajectories of sleep problems and sleep duration over time. Two main models were constructed: Model 1) adjusted for child’s sex, age and ethnic origin; and Model 2) additionally adjusted for maternal smoking during pregnancy, parity, and gestational age at birth. To test potential underlying mechanisms, two additional models were constructed both building on model 2: Model 3) additionally adjusted for postnatal depressive symptoms of the mother (as potential postnatal mediator) and father; and Model 4) testing effect modification, i.e., an interaction between prenatal stress and PRS_insomnia_ (in sleep problems analyses), or with PRS_Sleep Duration_ (sleep duration analyses). Two sensitivity analyses were also performed to assess potential selection bias. First, we reran all analyses in a sample with complete outcome data (n = 2,598), to evaluate whether the multiple imputation technique influenced the results. Second, main analyses were rerun in the subsample with available genetics data (n = 2,063), to evaluate the consistency and whether findings on gene-by-environment interaction can be generalized to the full sample.

## Results

### Sample Characteristics

Our sample was evenly spread across the sexes (50.7% girls), and 71.5% of the population was of Dutch origin. Largest proportion of the mothers were highly educated, and their depressive symptoms’ score at three years postpartum ranged between 0 and 3.66 (mean = 0.12, SD = 0.31). Children’s sleep duration decreased from 14.7 ± 3.0 h per 24 h at 2 months to 12.6 ± 1.1 at 3 years of age. Sleep problems also declined over time (Table 1). The sample included in this study was more likely to be Dutch (66% vs. 38%, p < 0.001) than those with no data on sleep outcomes, and mothers of the included children were more likely to highly educated (51.0% vs. 34.6%, p < 0.001) and experienced less stress during pregnancy (4.4 ± 3.8 vs. 6.6 ± 4.7, p < 0.001).

**Table 1:** Sample Characteristics

Characteristic (N = 4,930)	Estimate
Sex, % girls	50.7
Ethnic Origin	
Dutch, %	71.5
Other-Western, %	9.2
Other-non Western, %	24.5
Gestational age at birth	39.9 (1.7)
Prenatal Stress Score	
Life Events	1.8 (1.5)
Contextual	0.9 (1.3)
Parental	0.5 (0.6)
Interpersonal	1.5 (2.2)
Sleep problems, score	
2 months	2.6 (1.8)
1.5 years	1.6 (1.9)
2 years	2.5 (2.2)
3 years	1.5 (1.8)
6 years	1.0 (1.5)
Sleep duration, hours	
2 months	14.7 (3.0)
2 years	13.4 (1.1)
3 years	12.6 (1.1)
Maternal Characteristics	
Educational Level	
Low, %	42.6
Medium & High, %	54.7
Depressive symptoms at 3 years postpartum, score	0.12 (0.31)
Parity, % first born	57.6
Partnership status, single %	8.4

Numbers represent percentages or mean (SD)

### Prenatal Stress and Sleep Problems Across Early Childhood

Higher total prenatal stress was associated with more sleep problems across all time points between 2 months and 6 years of age (Table 2). Standardized effect estimates ranged between 0.21 (95%CI: 0.14 – 0.27) at 2 months to 0.45 (95%CI: 0.38;0.53) at 2 years, indicating small associations that persist over time. Associations between prenatal stress and sleep problems were present across different domains of stress (life events, contextual, parental and interpersonal stressors) and child’s sleep at different ages, with the exception of parental stressors (e.g., teenage or single parenthood) which were not associated with sleep problems at 2 months (B = 0.18, 95%CI: -0.02;0.10), but were related to more sleep problems from 1.5 years onwards. Longitudinal models, however, indicated that prenatal stress did not have an effect on the trajectory of sleep problems across time (Online Resource 2: Supplement Table 1, interactions between prenatal stress (domain) scores and age on sleep problems all p > 0.05). Controlling for additional covariates in Model 2 or for postnatal depressive symptoms of the mother and father in Model 3 did not materially change the effect estimates in any model (data not shown).

**Table 2:** Association between prenatal stress and mother reported sleep problems (n = 4,930)

**Mother reported sleep problems**										
	@ 2 months		@ 1.5 years		@ 2 years		@ 3 years		@6 years	
	B	P	B	P	B	P	B	P	B	P
	(95% CI)		(95% CI)		(95% CI)		(95% CI)		(95% CI)	
Prenatal Stress, total score	0.21	< 0.001	0.32	< 0.001	0.45	< 0.001	0.37	< 0.001	0.32	< 0.001
	(0.14;0.27)		(0.25;0.38)		(0.38;0.53)		(0.30;0.43)		(0.27;0.37)	
Life Events	0.1	0.001	0.2	< 0.001	0.27	< 0.001	0.26	< 0.001	0.19	< 0.001
	(0.04;0.16)		(0.14;0.26)		(0.20;0.34)		(0.20;0.34)		(0.14;0.24)	
Contextual Stress	0.17	< 0.001	0.2	< 0.001	0.28	< 0.001	0.22	< 0.001	0.18	< 0.001
	(0.11;0.23)		(0.14;0.26)		(0.20;0.34)		(0.16;0.28)		(0.13;0.23)	
Parental Stress	0.04	0.176	0.26	< 0.001	0.34	< 0.001	0.2	< 0.001	0.23	< 0.001
	(-0.02;0.10)		(0.19;0.32)		(0.27;0.41)		(0.13;0.26)		(0.18;0.28)	
Interpersonal Stress	0.18	< 0.001	0.23	< 0.001	0.35	< 0.001	0.28	< 0.001	0.25	< 0.001
	(0.12;0.22)		(0.16;0.29)		(0.28;0.41)		(0.22;0.34)		(0.21;0.30)	

*Models are adjusted for sex, age at sleep assessment and ethnic origin of the child. Every estimate represents a separate regression analysis

**Fig. 1:**
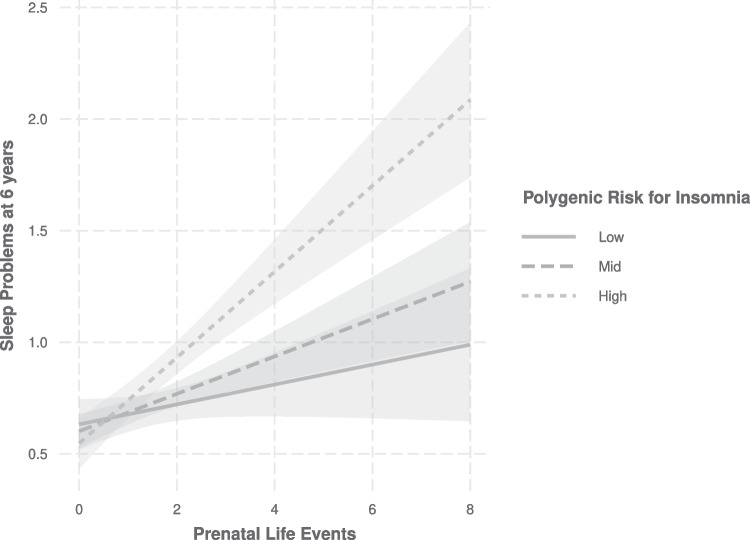
Polygenic Risk Score (PRS) for Insomnia modifies the association between prenatal life events and sleep problems at 6 years Shown are associations between prenatal life events (PLE) and sleep problems at 6 years (SP6) across levels of PRS for Insomnia. X-axis shows values of the predictor (PLE), Y-axis shows predicted values for SP6 (range 0–9) based on a regression model adjusted for age, sex and ethnicity. PRS Insomnia groups were defined as Low, 25^th^ Percentile (n = 516); High, 75^th^ percentile (n = 515); and Mid (n = 1,033) PRS values in between

A trend-level interaction between prenatal stress and PRS_insomnia_ on sleep problems at 6 years of age (B_interaction_ = 0.05, 95%CI: -0.002;0.108, p = 0.059) was present, indicating that polygenic liability for insomnia may exacerbate associations of prenatal stress on childhood sleep. Domain-specific analyses revealed that this was most prominent for the subdomain of prenatal life events, such that in children with a higher genetic risk for insomnia, prenatal life events had stronger associations with sleep problems at 6 years of age (B_interaction_ = 0.07, 95%CI: 0.02;0.13, p = 0.011) as compared to children with lower genetic risk for insomnia (Fig. 1).

### Prenatal Stress and Sleep Duration Across Early Childhood

We observed no evidence for an interaction between prenatal stress (either total score or domain specific) and PRS-SD at any time point. Total prenatal stress score was associated with shorter sleep duration at 2 months (B_hrs_ = -0.22, 95%CI: -0.32;-0.12), and at 2 years (B_hrs_ = -0.04, 95%CI -0.07; -0.001), but not with sleep duration at 3 years (B_hrs_ = 0.02, 95%CI: -0.02;0.06). Domain specific analyses (Table 3) revealed that prenatal stress across the different domains was associated with sleep duration at 2 months, whereas associations at 2 years seem to be cumulative; i.e. only the combined, but not the individual prenatal stress domains were significantly associated with sleep duration at 2 years of age (Table 3). In contrast, we observed an association between higher prenatal parental stress and longer sleep duration at 3 years (B_hrs_ = 0.05, 95%CI: 0.01;0.09). Prenatal stress was not associated with nighttime sleep only at 2 months and 2 years (Online Resource 3: Supplement Table 2), but was positively associated with nighttime sleep at 3 years (in particular parental and interpersonal stress). Longitudinal models indicated that higher prenatal stress across all domains was associated with a slower decrease in the developmental sleep duration across time (Online Resource 4: Supplement Table 3, B_total prenatal score_ = -0.06, 95%CI: -0.08;-0.04, B_age_ = -0.07, 95%CI: -0.07,-0.06, B_total prenatal stress*age_ = 0.002, 95%CI: 0.001;0.003, p < 0.001).

**Table 3:** Association between prenatal stress and mother reported sleep duration (n = 4,930)

	**Mother reported sleep duration**					
	@ 2 months		@ 2 years		@ 3 years	
	B (95% CI)	P	B (95% CI)	P	B (95% CI)	P
Prenatal Stress, total score	-0.22 (-0.32;-0.12)	< 0.001	-0.04 (-0.07;-0.001)	0.044	0.02 (-0.02;0.06)	0.255
Life Events	-0.21 (-0.31;-0.10)	< 0.001	-0.02 (-0.06;0.01)	0.126	0.03 (-0.01;0.07)	0.128
Contextual Stress	-0.11 (-0.22;-0.002)	0.045	-0.02 (-0.06;0.01)	0.185	-0.001 (-0.04;0.04)	0.946
Parental Stress	-0.24 (-0.34;-0.13)	< 0.001	-0.01 (-0.05;0.01)	0.262	0.05 (0.01;0.09)	0.009
Interpersonal Stress	-0.11 (-0.21;-0.02)	0.019	-0.03 (-0.06;0.01)	0.152	0.009 (-0.03;0.05)	0.649

*Models are adjusted for sex, age at sleep assessment and ethnic origin of the child

### Sensitivity Analyses

Results from analyses in the subsample with full observation (no missing values on sleep outcomes at any time point) are shown in Online Resource 5: Supplementary Table 4 (Sleep Problems) and Online Resource 6: Supplementary Table 5 (Sleep Duration). These are largely comparable with the main analyses, though effect estimates were smaller. Results in the subsample with genetic data were similar to the main results, although with slightly smaller effect estimates (see Online Resource 7 and 8).

## Discussion

With this study we showed robust associations of a stressful prenatal environment with poor sleep across early childhood. Specifically, prenatal stressors across different domains are associated with more insomnia-like sleep problems. Albeit small, these associations are persistent across the first 6 years of life, but the trajectory of sleep problems during this period does not seem to be affected. Additionally, we found evidence that prenatal stress, and in particular negative life events during the pregnancy interact with genetic liability for insomnia to exacerbate sleep problems at six years of age. Our results also indicate that sleep duration in infancy (two months) may be more liable to negative influences of a stressful prenatal environment as compared to sleep duration in toddlerhood (two or three years of age), and these associations do not vary across different levels of genetic liability for sleep duration. Prenatal stress, however, seems to be associated with a slower decrease in sleep duration in the first three years of life.

Our findings are largely in line with previous research showing associations between stress during pregnancy and more sleep problems in infancy (Morales-Muñoz et al., 2018) and early childhood (Simcock et al., 2019; van den Heuvel et al., 2021). Effect sizes are comparably small to moderate across all studies (0.02–0.04), and do not seem to vary meaningfully across age. In line with this, we did not observe any associations of prenatal stress with trajectories of sleep problems up to age six, contrary to a study showing a slower decrease in sleep problems across the first eleven years (Ksinan Jiskrova et al., 2021) in children exposed to more prenatal stress. These findings may suggest that associations of prenatal stress on sleep problems are persistent over time (i.e., stressful prenatal environment is related to more sleep problems throughout childhood, but not to a patterns of increase in sleep problems over time). The results may also reflect stability of stress from the prenatal to postnatal period, that in turn influences concurrent sleep quality. Accounting for postnatal psychopathology of the mother and father, however, did not alter these results. Alternatively, differences in the observed results may be related to the assessment of stress via subjective stress questionnaires vs. prospective assessment of the occurrence of stressors across pregnancy in our study (Chatterjee et al., 2018). Future studies should aim to incorporate both subjective and objective aspects of stress*.*

Fewer studies assessed the associations between prenatal stress and sleep duration, mostly reporting null findings in infancy, toddlerhood and early childhood (Chatterjee et al., 2018; Morales-Muñoz et al., 2018; O'Connor et al., 2007). In individual timepoint analyses, we observed negative associations of prenatal stress with infant sleep duration, but these associations seemed to attenuate at age two and diminished by age three years. This is likely due to the fact that typically seen decrease in sleep duration across the childhood years was less pronounced in those exposed to stressful prenatal environment. These findings suggest that the negative associations of a stressful prenatal environment on sleep duration might diminish or be taken over by the postnatal environment at later ages. Prenatal stress, however, may also shift the developmental decreasing trajectory of sleep duration in the first years of life resulting in a slower or delayed decrease in sleep duration. This is particularly important as children who follow a faster developmental decline in sleep duration are shown to have more advanced cognitive development as compared to children whose sleep duration decreases at a slower pace (Bernier et al., 2021).

We found prenatal stress across different domains to be associated with sleep problems up to age six and with infant sleep duration. At the age of two years, however, associations of prenatal stress on sleep duration tend to be cumulative, i.e., individual prenatal stressor domains are not associated with shorter sleep, whereas their cumulative effect (i.e., total prenatal stress score) is related to a slightly shorter sleep duration. This could be explained by increasing time lag from the exposure to specific stressors which in turn may be taken over by cumulative effect of postnatal influences.

Our study is, to our best knowledge, the first one to show that a stressful prenatal environment interacts with polygenic risk for poor sleep to shape children’s sleep. Interestingly, our results indicate that prenatal stress, and negative life events in particular, interact with genetic liability for insomnia to exacerbate sleep problems at age 6 years, but not at earlier ages. This is in line with previous research in twins showing increasing heritability of insomnia with age, whereas environmental effects tend to attenuate with age (Barclay et al., 2021). Our data further indicate that associations of prenatal stress with sleep duration do not vary across levels of genetic liability for sleep duration at any age. Taken together, these findings indicate that the prenatal environmental and genetic influences may act jointly to shape qualitative aspects of sleep, but not sleep duration during early childhood.

Several other mechanisms may play a role in the observed associations. Stress hormones (including cortisol) cross the placenta, which may adversely influence the development of the fetal stress-response system, and in turn predispose children to dysregulated sleep (Kim et al., 2015). Alternatively, stress-related maternal inflammation, changes in placental function, and epigenetic mechanisms have also been noted as adverse influences on feta neurodevelopment, including sleep (Babenko et al., 2015; Krontira et al., 2020; Lo Martire et al., 2020; Palagini et al., 2015). In addition to the pathophysiological pathways, maternal health behaviors during pregnancy may also play a role in child sleep disturbances (De Weerth, 2018). Stress in pregnancy has been associated with health impairing behaviors (e.g. smoking), and mental health problems, that are known risk factors for poor childhood sleep (Stone et al., 2010). Similarly, fathers’ health, behavior and lifestyle could also influence maternal experience of stress, or may have a direct link to sleep regulation in the offspring. These prenatal, perinatal and postnatal factors (smoking during pregnancy, gestational age at birth, psychopathology of the mother and father), however, did not account for the observed effects in our analyses. Future studies should aim to disentangle these possible pathways, both at biological level (epigenetics, neurobiology, stress response system) and at behavioral level (maternal and paternal prenatal and postnatal lifestyle). Our results could also be explained by the persistence of stressors in postnatal life. In other words, postnatal stress which was present during pregnancy and persisted after birth may have a direct negative influence on childhood sleep (Sharp et al., 2019). Accounting for postnatal stress, however, would introduce inaccuracy in the effect estimates as these variables are inherently correlated and postnatal factors are likely on the pathway from prenatal stress to children’s sleep. In addition, future studies should also study if timing of experienced stress during the pregnancy may have differential effects on sleep in the offspring (e.g., stress experienced in the first trimester may be more detrimental to sleep regulation that stress experienced later in the pregnancy).

Our study has several limitations, including the potential information bias, owing to mother reports at both ends of the associations, and selection bias, owing to nonresponse and/or lost-to follow-up. Imputing missing values of predictors and outcomes should, however, minimize selective follow up and thus reduce selection bias (van Ginkel et al., 2020). Our measure of sleep duration may have introduced error, as it was an estimated total hours of sleep based on both continuous and categorical measures. In addition, analyses using polygenic risk scores were performed in a subsample of European Ancestry, which limits the generalization of the observed gene-by-environment interaction to other populations (Ma et al., 2021). While we adjusted our models hierarchically for several perinatal and postnatal factors that may influence child’s sleep, unmeasured confounders, and in particular postnatal factors may still explain some of our findings. Future longitudinal studies with longer follow-up should take time-varying postnatal factors into account to assess how enduring these associations are. Our study, however, also has some important strengths including the large sample size largely representative of the general pediatric population, prospective assessment of multiple domains of stress across the pregnancy and repeatedly measured sleep across early childhood.

In conclusion, our study highlights the importance of the prenatal environment in shaping sleep regulation. Our data further suggest that associations of prenatal stress with poor childhood sleep are not transient. Importantly, we also show that prenatal stress interacts with genetic predisposition for poor sleep to aggravate childhood sleep problems. Associations of the prenatal environment with sleep duration, in turn, seem to be more pronounced in infancy, and may attenuate at later ages. If replicated, these findings might inform early detection and intervention programs that target sleep in children from high-risk pregnancies.

## Contributions

All authors contributed to the study conception and design. The first draft of the manuscript was written by D. Kocevska and all authors critically evaluated the manuscript. Dr. Kocevska ran the analyses with close collaboration with I. Schuurmans. A.I. Luik supervised the work and together with other senior authors (E.J.W. van Someren, C..M. Cecil, P.W. Jansen) co-drafted and critically reviewed the manuscript. All authors approved the final manuscript as submitted and agree to be accountable for all aspects of the work.

### Supplementary Information

Below is the link to the electronic supplementary material.

Supplementary file1 (DOCX 37 KB)

## Data Availability

Data from the analyses is available only upon official request to Data Managment of the Generation R, and signing relevant confidentiality agreement and corresponding costs.
