# Supplementary material for: A Longitudinal Study of Stress During Pregnancy, Children’s Sleep and Polygenic Risk for Poor Sleep in the General Pediatric Population
Source: Res Child Adolesc Psychopathol. 2023 Jul 13;51(12):1909–18. doi: 10.1007/s10802-023-01097-2 (PMC10661881; doi:10.1007/s10802-023-01097-2)
Supplement: Supplementary file 1 — Supplementary file1 (DOCX 37 KB) [file 10802_2023_1097_MOESM1_ESM.docx]

Supplementary Information

A Longitudinal Study of Stress During Pregnancy, Children’s Sleep and Polygenic Risk for Poor Sleep in the General Pediatric Population

Desana Kocevska^1,2,3^, Isabel K. Schuurmans^3,4^, Charlotte A.M. Cecil^2,3,4^, Pauline W. Jansen^2,3,5^, Eus J.W. van Someren^1,6,7^, Annemarie I. Luik^2,4^

^1^Department of Sleep and Cognition, Netherlands Institute for Neuroscience, Amsterdam, Netherlands;
^2^Department of Child and Adolescent Psychiatry/Psychology, Erasmus MC University Medical Center, Rotterdam, Netherlands;
^3^Generation R Study, Erasmus MC University Medical Center Rotterdam, Rotterdam, Netherlands;

^4^Department of Epidemiology, Erasmus MC University Medical Center Rotterdam, Netherlands;
^5^Department of Psychology, Education, and Child Studies, Erasmus University Rotterdam, Rotterdam, The Netherlands.
^6^Department of Psychiatry, Amsterdam Public Health Research Institute and Amsterdam Neuroscience research institute, Amsterdam UMC, Vrije Universiteit, Netherlands;
^7^Department of Integrative Neurophysiology, Center for Neurogenomics and Cognitive Research, Amsterdam Neuroscience, Vrije Universiteit Amsterdam, Netherlands

Journal: Research on Child and Adolescent Psychopathology

Correspondence to: D. Kocevska (d.kocevska@erasmusmc.nl)

**Online Recourse 1: Supplementary Methods**

|  | | | | |
| --- | --- | --- | --- | --- |
|  | Valid range | Instrument | Time Points | Items; risk |
| **Life events** | 0.00-15.00 | Social Readjustment Rating Scale | 20-25w gestation | Your partner or child died; yes |
|  |  |  |  | A friend or relative died; yes |
|  |  |  |  | Child, partner or relative was seriously ill; yes |
|  |  |  |  | You lost your job; yes |
|  |  |  |  | You had problems at work or school; yes |
|  |  |  |  | You moved house; yes |
|  |  |  |  | Your house or car was burgled; yes |
|  |  | 12-Item Short Form Health | 20-25w gestation | You were ill (moderate or poor health); yes |
|  |  | General questionnaire | 12-20w gestation | This pregnancy was unplanned; yes |
|  |  |  | 20-25w gestation | You experienced vaginal bleeding; yes |
|  |  |  | 30w gestation | You were admitted to hospital (>24 hours); yes |
|  |  |  |  | You had a test to see if your baby was abnormal; yes |
|  |  |  |  | You were unhappy about the obstetric care; yes |
|  |  | Pregnancy Outcome questionnaire | 12-20w gestation | You were often worried about the health of the baby; yes |
|  |  |  |  | You were often worried about the pregnancy; yes |
| **Contextual stress** | 0.00-6.00 | Social Readjustment Rating Scale | 20-25w gestation | Your income was reduced; yes |
|  |  | Long lasting difficulties questionnaire | 20-25w gestation | Housing adequacy (e.g., too small, lack of privacy); yes |
|  |  |  |  | You had a major financial problem; yes |
|  |  | Material deprivation | 30w gestation | Housing basic living (inadequate heating, no washing machine, or no refrigerator); yes |
|  |  |  |  | Housing defects (cold or draught, windows are damp inside, or walls or furniture are damp); yes |
|  |  |  |  | Financial difficulties ; yes |
| **Parental stress** | 0.00-6.00 | Brief symptom inventory | 20-25w gestation | Psychopathology of mother, > 0.71 |
|  |  | General questionnaire | Enrollment | Early parenthood; age mother <19 yrs. |
|  |  |  |  | Education main caregiver; < phase 2 (higher) secondary education |
|  |  |  | 20-25w gestation | You were in trouble with the law; yes |
|  |  |  |  | Violence offence; yes |
|  |  |  |  | Public order offence; yes |
| **Interpersonal stress** | 0.00-18.00 | Long lasting difficulties questionnaire | 20-25w gestation | You argued with your partner; yes |
|  |  |  |  | You had arguments with your family or friends; yes |
|  |  |  |  | Difficulties in contact with others; yes |
|  |  | General questionnaire | Enrollment | Marital status: single |
|  |  |  | 20-25w gestation | Family size; > 3 persons |
|  |  | Social Readjustment Rating Scale | 20-25w gestation | You were divorced; yes |
|  |  | Family assessment device | 20-25w gestation | Family affection problems; yes |
|  |  |  |  | Difficulty in making plans; yes |
|  |  |  |  | Disapproval of other; yes |
|  |  |  |  | Difficulty in talking about sadness; yes |
|  |  |  |  | Avoidance of talking about problems; yes |
|  |  |  |  | Feelings of being unaccepted; yes |
|  |  |  |  | Unpleasant and painful feelings; yes |
|  |  |  |  | Inability to solve problems; yes |
|  |  |  |  | Decision-making is a problem; yes |
|  |  |  |  | Distrust of each other; yes |
|  |  |  |  | Conflicts with each other; yes |
|  |  |  |  | Family support problems; yes |

**Online Recourse 2: Supplement Table 1. Longitudinal association between prenatal stress and mother reported sleep problems between 2 months and 6 years**

|  | | | | | | | | |
| --- | --- | --- | --- | --- | --- | --- | --- | --- |
|  | Main Effect | |  | Slope | |  | Main effect*Slope | |
|  | B (95% CI) | P |  | B (95% CI) | P |  | B (95% CI) | P |
| Prenatal Stress, total score | 0.07 (0.06;0.09) | <0.001 |  | -0.02 (-0.03;-0.02) | <0.001 |  | 0.00002 (-0.0002;0.0003) | 0.844 |
| Life Events | 0.15 (0.11;0.19) | <0.001 |  | -0.02 (-0.02;-0.02) | <0.001 |  | 0.0001 (-0.001;0.001) | 0.683 |
| Contextual Stress | 0.18 (0.14;0.23) | <0.001 |  | -0.02 (-0.02;-0.02) | <0.001 |  | -0.001 (-0.001;0.0004) | 0.277 |
| Parental Stress | 0.32 (0.24;0.41) | <0.001 |  | -0.02 (-0.02;-0.02) | <0.001 |  | 0.001 (-0.001;0.003) | 0.237 |
| Interpersonal Stress | 0.10  (0.08;0.13) | <0.001 |  | -0.03 (-0.03;-0.02) | <0.001 |  | 0.0001 (-0.0004;0.0001) | 0.810 |

Models are adjusted for sex, age at sleep assessment and ethnic origin of the child. Effect estimates are based on a linear mixed model. Main effects represent effects of prenatal stress (domains) scores on repeatedly measured sleep problems; slope represents the effect of age on sleep problems, and the interaction (main effect * slope) represents effect of prenatal stress (domain) scores on changes of sleep problems across age.

**Online Recourse 3: Supplement Table 2. Association between prenatal stress and mother reported sleep duration during the night (n=4,930)**

|  | **Mother reported sleep duration** | | | | | | | |  |
| --- | --- | --- | --- | --- | --- | --- | --- | --- | --- |
|  | @ 2 months |  |  | @ 2 years |  |  | @ 3 years |  | |
|  | B (95% CI) | P |  | B (95% CI) | P |  | B (95% CI) | P | |
| Prenatal Stress, total score | -0.03 (-0.02;0.04) | 0.325 |  | 0.01 (-0.02;0.04) | 0.501 |  | 0.04 (0.01;0.06) | 0.003 | |
| Life Events | -0.04 (-0.10;0.02) | 0.194 |  | -0.01 (-0.04;0.02) | 0.381 |  | -0.001 (-0.02;0.02) | 0.863 | |
| Contextual Stress | -0.02 (-0.08;0.04) | 0.532 |  | 0.02 (-0.01;0.06) | 0.138 |  | 0.02 (-0.01;0.04) | 0.171 | |
| Parental Stress | -0.04 (-0.10;0.02) | 0.231 |  | 0.04 (0.01;0.08) | 0.008 |  | 0.09 (0.06;0.11) | <0.001 | |
| Interpersonal Stress | -0.01 (-0.08;0.06) | 0.762 |  | 0.003 (-0.02;0.03) | 0.841 |  | 0.03 (0.01;0.06) | 0.005 | |

*Models are adjusted for sex, age at sleep assessment and ethnic origin of the child

**Online Recourse 4: Supplement Table 3. Longitudinal association between prenatal stress and mother reported sleep duration between 2 months and 3 years**

|  | | | | | | | | |
| --- | --- | --- | --- | --- | --- | --- | --- | --- |
|  | Main Effect | |  | Slope | |  | Main effect*Slope | |
|  | B (95% CI) | P |  | B (95% CI) | P |  | B (95% CI) | P |
| Prenatal Stress, total score | -0.06 (-0.08;-0.04) | <0.001 |  | -0.07 (-0.07;-0.06) | <0.001 |  | 0.002 (0.001;0.003) | <0.001 |
| Life Events | -0.16 (-0.22;-0.11) | <0.001 |  | -0.07 (-0.07;-0.06) | <0.001 |  | 0.01 (0.004;0.008) | <0.001 |
| Contextual Stress | -0.12 (-0.18;-0.05) | 0.001 |  | -0.07 (-0.07;-0.06) | <0.001 |  | 0.004 (0.002;0.007) | <0.001 |
| Parental Stress | -0.43 (-0.18;-0.05) | 0.001 |  | -0.07 (-0.07;-0.06) | <0.001 |  | 0.004 (0.002;0.01) | <0.001 |
| Interpersonal Stress | -0.06 (-0.09;-0.03) | <0.001 |  | -0.07 (-0.07;-0.06) | <0.001 |  | 0.002 (0.001;0.003) | <0.001 |

Models are adjusted for sex, age at sleep assessment and ethnic origin of the child. Effect estimates are based on a linear mixed model. Main effects represent effects of prenatal stress (domains) scores on repeatedly measured sleep duration; slope represents the effect of age on sleep duration, and the interaction (main effect * slope) represents effect of prenatal stress (domain) scores on changes of sleep duration across age.

**Online Recourse 5: Supplement Table 4. Association between prenatal stress and mother reported sleep problems, in sample with complete observations (n=2,598)**

|  |  |  |  |  | **Mother reported sleep problems** | | | | | | | | | | | | |  |
| --- | --- | --- | --- | --- | --- | --- | --- | --- | --- | --- | --- | --- | --- | --- | --- | --- | --- | --- |
|  | @ 2 months | | | | |  |  | @ 1.5 years |  |  | @ 2 years |  |  | @ 3 years |  |  | @6 years |  |
|  | B (95% CI) | | | | | P |  | B (95% CI) | P |  | B (95% CI) | P |  | B (95% CI) | P |  | B (95% CI) | P |
| Prenatal Stress, total score | 0.18 (0.11;0.25) | | | | | <0.001 |  | 0.29 (0.22;0.37) | <0.001 |  | 0.36 (0.28;0.44) | <0.001 |  | 0.33 (0.26;0.40) | <0.001 |  | 0.27 (0.22;0.33) | <0.001 |
| Life Events | 0.08 (0.01;0.15) | | | | | 0.021 |  | 0.18 (0.11;0.26) | <0.001 |  | 0.25 (0.17;0.33) | <0.001 |  | 0.22 (0.16;0.30) | <0.001 |  | 0.17 (0.11;0.23) | <0.001 |
| Contextual Stress | 0.13 (0.05;0.19) | | | | | 0.001 |  | 0.19 (0.12;0.26) | <0.001 |  | 0.24 (0.17;0.32) | <0.001 |  | 0.20 (0.12;0.26) | <0.001 |  | 0.15 (0.10;0.21) | <0.001 |
| Parental Stress | -0.03 (-0.09;0.05) | | | | | 0.488 |  | 0.21 (0.13;0.28) | <0.001 |  | 0.24 (0.17;0.32) | <0.001 |  | 0.16  (0.10;0.23) | <0.001 |  | 0.19 (0.13;0.24) | <0.001 |
| Interpersonal Stress | 0.18 (0.10;0.25) | | | | | <0.001 |  | 0.22 (0.14;0.29) | <0.001 |  | 0.25 (0.17;0.32) | <0.001 |  | 0.26 (0.19;0.33) | <0.001 |  | 0.21 (0.16;0.27) | <0.001 |

*Models are adjusted for sex, age at sleep assessment and ethnic origin of the child

| **Online Recourse 6: Supplement Table 5. Association between prenatal stress and mother reported Sleep Duration, in sample with complete observations (n=2,598)** | | | | | | | | | | | | |  |
| --- | --- | --- | --- | --- | --- | --- | --- | --- | --- | --- | --- | --- | --- |
|  |  |  |  |  | **Mother reported sleep duration** | | | | | | | |  |
|  | @ 2 months | | | | |  |  | @ 2 years |  |  | @ 3 years |  | |
|  | B (95% CI) | | | | | P |  | B (95% CI) | P |  | B (95% CI) | P | |
| Prenatal Stress, total score | -0.25 (-0.37;-0.13) | | | | | <0.001 |  | -0.06 (-0.10;-0.01) | 0.009 |  | 0.003 (-0.04;0.05) | 0.884 | |
| Life Events | -0.25 (-0.37;-0.10) | | | | | <0.001 |  | -0.04 (-0.08;0.004) | 0.073 |  | 0.01 (-0.04;0.004) | 0.737 | |
| Contextual Stress | -0.09 (-0.21;0.003) | | | | | 0.137 |  | -0.04 (-0.08;0.01) | 0.095 |  | -0.004 (-0.05;0.04) | 0.839 | |
| Parental Stress | -0.23 (-0.34;-0.11) | | | | | <0.001 |  | -0.04 (-0.08;0.01) | 0.098 |  | 0.03 (-0.01;0.16) | 0.164 | |
| Interpersonal Stress | -0.14 (-0.26;-0.02) | | | | | 0.018 |  | -0.04 (-0.08;0.001) | 0.058 |  | -0.01 (-0.05;0.04) | 0.818 | |

*Models are adjusted for sex, age at sleep assessment and ethnic origin

**Online Recourse 7: Supplement Table 6. Association between prenatal stress and mother reported sleep problems, in sample with genetic data (n=2063)**

|  |  |  |  |  | | **Mother reported Sleep Problems** | | | | | | | | | | | |  |
| --- | --- | --- | --- | --- | --- | --- | --- | --- | --- | --- | --- | --- | --- | --- | --- | --- | --- | --- |
|  | @ 2 months | | | |  | |  | @ 1.5 years |  |  | @ 2 years |  |  | @ 3 years |  |  | @6 years |  |
|  | B (95% CI) | | | | P | |  | B (95% CI) | P |  | B (95% CI) | P |  | B (95% CI) | P |  | B (95% CI) | P |
| Prenatal Stress, total score | 0.05 (-0.03;0.13) | | | | 0.129 | |  | 0.14 (0.07;0.23) | <0.001 |  | 0.29 (0.20;0.37) | <0.001 |  | 0.24 (0.16;0.32) | <0.001 |  | 0.25 (0.19;0.32) | <0.001 |
| Life Events | 0.01 (-0.07;0.09) | | | | 0.801 | |  | 0.10 (0.02;0.19) | 0.012 |  | 0.17 (0.10;0.26) | <0.001 |  | 0.14 (0.06;0.22) | 0.001 |  | 0.15 (0.09;0.21) | <0.001 |
| Contextual Stress | 0.06 (-0.03;0.14) | | | | 0.181 | |  | 0.08 (-0.01;0.16) | 0.066 |  | 0.17 (0.09;0.26) | <0.001 |  | 0.17 (0.09;0.25) | <0.001 |  | 0.17 (0.11;0.23) | <0.001 |
| Parental Stress | -0.12 (-0.19;0.03) | | | | 0.004 | |  | 0.15 (0.07;0.23) | <0.001 |  | 0.19 (0.12;0.23) | <0.001 |  | 0.09  (0.004;0.16) | 0.037 |  | 0.13 (0.08;0.19) | <0.001 |
| Interpersonal Stress | 0.07 (-0.01;0.15) | | | | 0.095 | |  | 0.09 (0.01;0.17) | 0.031 |  | 0.21 (0.12;0.29) | <0.001 |  | 0.18 (0.11;0.27) | <0.001 |  | 0.19 (0.13;0.26) | <0.001 |

*Models are adjusted for sex, age at sleep assessment and ethnic origin

**Online Recourse 8: Supplement Table 7. Association between prenatal stress and mother reported sleep duration, in sample with genetic data (n=2063)**

|  |  |  |  |  | **Mother reported Sleep Duration** | | | | | | | |  |  |
| --- | --- | --- | --- | --- | --- | --- | --- | --- | --- | --- | --- | --- | --- | --- |
|  | @ 2 months | | | | |  |  | @ 2 years |  |  | @ 3 years |  | |  |
|  | B (95% CI) | | | | | P |  | B (95% CI) | P |  | B (95% CI) | P | |  |
| Prenatal Stress, total score | -0.19 (-0.32;0.05) | | | | | 0.032 |  | -0.01 (-0.05;-0.04) | 0.800 |  | 0.02 (-0.06;0.05) | 0.773 | | |
| Life Events | -0.16 (-0.30;-0.04) | | | | | 0.013 |  | 0.03 (-0.02;0.08) | 0.216 |  | 0.03 (-0.01;0.09) | 0.191 | | |
| Contextual Stress | -0.10 (-0.25;-0.05) | | | | | 0.185 |  | -0.01 (-0.05;0.04) | 0.822 |  | -0.02 (-0.07;0.03) | 0.503 | | |
| Parental Stress | -0.14 (-0.27;0.002) | | | | | 0.054 |  | -0.02 (-0.07;0.02) | 0.325 |  | 0.03 (-0.02;0.09) | 0.206 | | |
| Interpersonal Stress | -0.10 (-0.24;0.03) | | | | | 0.124 |  | -0.02 (-0.06;0.03) | 0.371 |  | -0.04 (-0.09;0.02) | 0.168 | | |
| Models are adjusted for sex, age at sleep assessment and ethnic origin | | | | | | | | | | | | | | |
